# The Quality of Health Towards Resilience Community in Indonesia: A Resilience Policy Discourse

**DOI:** 10.1002/hsr2.71324

**Published:** 2025-10-03

**Authors:** Abdillah Abdillah, Siti Sofiaturrohmah, Prakoso Bhairawa Putera, Danilo V. Rogayan

**Affiliations:** ^1^ Gradute Program in Administrative Science, Faculty of Social and Political Sciences Universitas Padjadjaran Bandung, Dago, Coblong, Kota Bandung, Jawa Barat Indonesia; ^2^ Center for Decentralization and Participatory Development Research, Faculty of Social and Political Sciences Universitas Padjadjaran Bandung, Dago, Coblong, Kota Bandung, Jawa Barat Indonesia; ^3^ Department of Government Studies, Faculty of Social and Political Sciences Universitas Padjadjaran Bandung, Dago, Coblong, Kota Bandung, Jawa Barat Indonesia; ^4^ Research Center for Public Policy, National Research and Innovation Agency (BRIN) Jakarta Selatan Indonesia; ^5^ Graduate School President Ramon Magsaysay State University Zambales Philippines

## Transparency Statement

All authors have read and approved the final version of the article. (Corresponding author or article guarantor) had full access to all of the data in this study and takes complete responsibility for the integrity of the data and the accuracy of the data analysis.

Dear Editor,

Recent research has suggested factors that influence vaccine acceptance during the COVID‐19 pandemic. However, research trends show that there is a real dearth of research on preferences and willingness to pay (WTP) among chronic disease (ChD) patients at high risk of severe COVID‐19 [1]. They argue that health authorities should prioritize high efficacy and long‐term protection in vaccine procurement, ensuring that neither is compromised.

In Indonesia, the impact of the COVID‐19 pandemic health crisis has had significant implications for domestic conditions that not only affect the quality of health and community welfare, but also affect the economy, governance, health service systems, and public behavior‐perception [2, 3]. This paper is a response to the evaluation of regulations and cooperation between central and local governments in facing the challenges of the COVID‐19 pandemic health crisis in Indonesia.

Based on the WHO and the Indonesian Ministry of Health report, the Government of Indonesia has made various responses [4, 5], including: (a) The Indonesian government has prioritized patients with comorbidities as a group prioritized for vaccination, especially in the initial wave of vaccination. But in its implementation, there are still administrative obstacles, such as the lack of data on chronic disease patients systematically registered at primary health care facilities; (b) Socialization to ChD patients regarding the urgency of vaccination is also still less mature; (c) In addition, proper policy review in health infrastructure development and poor governance such as health funding mechanisms and various overlapping roles between local and national authorities are challenges that need to be reviewed in realizing quality health for community resilience in Indonesia [6, 7].

However, it should be noted that realizing the quality of health for community resilience against the threat of the Covid‐19 pandemic health crisis, it is not enough to pay attention to policy initiatives, service governance, cross‐sectoral cooperation, adequate funding and health infrastructure, or the provision of other health support facilities that have been widely done [3, 6, 7]. It is necessary to pay attention to various dimensions of social, economic‐community, psychological aspect (mental health), institutional, and infrastructure resilience to ensure appropriate policy initiatives, sufficient funding, inclusive and needed health services to harmonious cooperation between each stakeholder [7] which can be done through attention to individuals in understanding health problems and appropriate handling. On the other hand, institutions/organizations need to provide governance schemes to sufficient funding in providing effective and beneficial health services for the community. In addition, the legal dimension needs to have the right policies and can fill the legal gap during the Covid‐19 pandemic in Indonesia [6, 7].

Lessons learned from the COVID‐19 pandemic in Indonesia in improving the quality of health for community resilience in facing the challenges of the Covid‐19 Pandemic health emergency: (a) building a governance resilience scheme in public health services; (b) the need for appropriate policy support and harmonious cooperation and collaboration between stakeholders; (c) the importance of public education campaigns aimed at uncovering mitigations about the disease and creating a supportive environment for health workers; and (d) the availability of adequate health facilities; to (e) encourage innovative and participatory handling models in improving the quality of health for community resilience.

In conclusion, the COVID‐19 pandemic in Indonesia has revealed the linkages between health, socio‐economics, governance, and public policy. Lessons learned from the challenges of the Covid‐19 pandemic health crisis in Indonesia underscore the urgency of resilience governance and policy discretion in strategies to improve the quality of health for community resilience as important steps in dealing with future health emergencies.

## Author Contributions

**Abdillah Abdillah:** conceptualization, investigation, funding acquisition, writing – original draft, methodology, validation, visualization, writing–review and editing, software, formal analysis, project administration, data curation, resources, supervision. **Siti Sofiaturrohmah:** writing – original draft, conceptualization, validation, writing–review and editing. **Prakoso Bhairawa Putera:** formal analysis, supervision, resources, data curation, validation. **Danilo V. Rogayan:** conceptualization, validation, supervision.

## Conflicts of Interest

The authors declare no conflicts of interest.

## Data Availability

The data set used to support the findings of this study is available from the corresponding author upon request.
